# First validation study of the living with long term conditions scale (LwLTCs) among English-speaking population living with Parkinson’s disease

**DOI:** 10.1186/s12955-023-02154-6

**Published:** 2023-07-10

**Authors:** Leire Ambrosio, Kelly Hislop-Lennie, Nestor Serrano-Fuentes, Corine Driessens, Mari Carmen Portillo

**Affiliations:** 1 School of Health Sciences and NIHR Applied Research Collaboration Wessex, University of Southampton, Hampshire, UK; 2 School of Health Sciences, University of Bournemouth, Dorset, UK; 3 NIHR Applied Research Collaboration Wessex, Hampshire, UK

**Keywords:** Living with long term conditions, Validation, Parkinson’s disease, Psychometric properties

## Abstract

**Introduction:**

Parkinson’s disease is the second most prevalent neurodegenerative disease, affecting 10 million people worldwide. Health and social care professionals need to have personalised tools to evaluate the process of living with Parkinson’s disease and consequently, plan individualised and targeted interventions. Recently, the English version of the Living with Long term conditions (LwLTCs) scale has been developed filling an important gap related to person-centred tools to evaluate the process of living with long term conditions among English-speaking population. However, no validation studies for testing its psychometric properties have been conducted.

**Aim:**

To analyse the psychometric properties of the LwLTCs scale in a wide English-speaking population living with Parkinson’s disease.

**Methods:**

Validation study, with an observational and cross-sectional design. The sample was composed of individuals living with Parkinson’s disease from non-NHS services in the community. Psychometric properties including feasibility and acceptability, internal consistency, reproducibility, and construct, internal and known-groups validity were tested.

**Results:**

A total sample of 241 people living with Parkinson’s disease were included. 6 individuals did not complete 1 or 2 items on the scale. Ordinal alpha was 0.89 for the total scale. The intraclass correlation coefficient for the total scale was 0.88. The LwLTCs scale is strongly correlated with scales measuring satisfaction with life (r_s_=0.67), quality of life (r_s_=0.54), and moderately correlated with social support (r_s_=0.45). Statistically significant difference just for therapy and co-morbidity, yet no for gender, employment situation, or lifestyle changes.

**Conclusions:**

The LwLTCs scale is a valid scale to evaluate how the person is living with Parkinson’s disease. Future validation studies to prove the repeatability of the total scale and particularly, domains 3-Self-management, and 4-Integration and internal consistency will be needed. Developing further studies on the English version of the LwLTC in people with other long term conditions is also proposed.

**Supplementary Information:**

The online version contains supplementary material available at 10.1186/s12955-023-02154-6.

## Background

Long term conditions (LTCs) have become one of the leading health related issues worldwide being responsible for 41 million deaths per year [1]. Among LTCs, neurodegenerative and progressive disorders, such as Parkinson’s disease (PD) stand out. PD is the second most prevalent neurodegenerative disease, affecting 10 million people worldwide [2]. More specifically, in Western countries PD has a prevalence between 108 and 250/100,000 and even higher for individuals over 65 years old, which could be up to 950/100,000 [3]. Medical costs for people with PD are twice those of people without PD. Hence, the annual costs per patient per year range from around £16,582 in the UK [4].

PD is a complex and disabling disorder characterized by a combination of motor signs, such as bradykinesia, rigidity, or tremor, but also non-motor symptoms like psychiatric disorders, pain, or sleep disturbance [2]. Throughout the PD course, individuals experience a progressive intensification of motor and non-motor symptoms resulting in an increasing limitation in the daily living [2]. Concretely, recent evidence conducted in PD population [3, 5, 6] showed that non-motor symptoms, have a significant and detrimental impact on the person’s daily living. In this regard, living with PD is comprised as a unique process for each person, influenced by factors such as social support, or satisfaction with life, affecting their quality of life and wellbeing [3, 7, 8]. For instance, Ambrosio and colleagues [7] identified that living with PD is strongly associated with social support and satisfaction with life. In this sense, highly perceived levels of social support and satisfaction with life, were strongly related to a positive living with PD. Similarly, additional studies conducted in PD population [3] highlighted the strong association between living with PD and quality of life, showing that a negative experience living with PD is associated with low self-esteem, or poor quality of life, among others. Therefore, considering personal factors related to the person’s daily living is important to achieve a positive living and promote a better quality of life and wellbeing [9]. In this context, health and social care professionals need to have a comprehensive understanding of how the person is living with PD to foster their needs and promote a positive living [3, 8]. Therefore, it is paramount to have holistic instruments to evaluate the process of living with the disease and, consequently, plan personalised, comprehensive and targeted interventions.

Currently, despite the great number of existing tools in clinical practice and research [11], there are no specific tools to evaluate the process of living with PD holistically. Currently, the Long-Term Conditions Questionnaire [12] is the only measure that evaluates a similar construct to ‘living with’. However, there is lack of understanding regarding the interpretation of punctuation and implementation of care pathway. Therefore, to our knowledge, the Living with LTCs (LwLTCs) scale is the only available instrument that evaluates how the person is living with a condition as PD, focusing on the process and capturing a more complete picture of the experience of living with LTCs, rather than just some patient outcomes [13]. The LwLTCs scale was originally developed in the Spanish language (named as *EC-PC: Escala de Convivencia con un Proceso Crónico*) and has achieved a wide and international trajectory in Spain and South America among different LTCs, such as PD [13, 14]. Concretely, results from the Spanish-speaking validation study in PD [13, 14] showed satisfactory psychometric properties (Cronbach’s alpha values ranged between 0.7 and 0.9 and internal validity correlations ranged from 0.5 to 0.8). Recently, the English version of the LwLTCs scale has been developed filling an important gap related to reliable and valid person-centred tools to evaluate the process of living with different LTCs among English-speaking population [15]. Preliminary psychometric properties of this new English person-centred tool showed satisfactory and promising results [15]. However, the LwLTCs scale is waiting for validation studies to test its psychometric properties in specific conditions such as PD among English-speaking population.

Therefore, the aim of this study is to analyse the psychometric properties of the LwLTCs scale in a wide English-speaking population living with PD.

## Methods

### Design

Validation study, with an observational and cross-sectional design.

This project has emerged from the international collaboration between NIHR Applied Research Collaboration Wessex (UK) and the ReNACE research programme (Spain), as a continuation of an existing international interdisciplinary network of researchers in the area of LTCs.

### Sample, sampling and sample size

A consecutive cases sampling frame [16, 17] was applied to participant identification until the required sample size of 5 to 10 participants per item was reached. In addition, sample size was based on estimating correlation to a suitable degree of precision; assuming a correlation of 0 (worst-case scenario in statistical terms), a sample size between 200 and 260 was ensuring a 95% confidence interval no wider than +/-0.12. We believed this was sufficiently narrow to judge the associations between the LwLTCs-PD scale and other included scales in this study. The following inclusion criteria were established: (1) having been diagnosed with PD by a GP or neurologist according to internationally recognised diagnostic criteria [18]; (2) being able to read, understand, and answer written questionnaires; (3) being fluent in speaking in English by understanding and answering in a conversation; and (4) being able to provide written informed consent. In the same way, the following exclusion criteria were ensured: (1) presenting cognitive deterioration and/or current psychiatric disorders, comorbidity or any other disorder that could interfere with or impede the reliability of the assessment of LTC manifestations or objectives of the study; and (2) not meeting the inclusion criteria.

The sample was composed of individuals living with PD from non-NHS services in the community. Recruitment was undertaken via voluntary organisations such as charity websites (i.e., Parkinson’s UK), and social media (i.e., Facebook and Twitter). Participants were self-selecting and confirmed that they met the inclusion/exclusion. Recruitment continued until a final sample size of 241 participants was reached.

### Instruments

The scale to be validated, the LwLTCs scale, is a self-reported measure containing 26 items grouped in five domains: 1-Acceptance (4 items), 2-Coping (7 items), 3-Self-management (4 items), 4-Integration (5 items) and 5-Adjustment (6 items). Answer categories range from 0 (nothing/never) to 4 (much/always). Higher domain scores indicate better living with the condition, as PD [13, 14]. Prior to this validation study, the scale was translated and cross-culturally adapted from the original LwLTCs scale (Spanish-language) to make it suitable for an English-speaking population [15]. The translation and cross-cultural adaptation process was conducted by a panel of four native English speaker experts. In addition, approval of the English version was sought from the original author of the LwLTC Scale in Spanish language [15].

Sociodemographic data such as age, gender, ethnicity, marital status, educational level, employment situation or household income were collected. Besides, PD related information such as the age of diagnosis, duration of PD, medication, surgery, and therapy for PD were collected. Mirroring previous validation studies carried out in PD population [13, 14], the following self-reported measuring scales were included in this study:

The Duke-UNC Functional Social Support Questionnaire (DUFSS) [19] includes 11 five-point Likert scale items measuring the level of social support for somebody living with a LTC. Higher DUFSS scores indicate higher levels of social support. The DUFSS presented adequate psychometric properties, showing a Cronbach’s alpha value of 0.9 and strong construct validity [20].

The World Health Organization Quality of Life Instrument-Brief (WHOQOL-BREF) [20] consists of 26 five-point Likert scale items measuring quality of life in the past 2 weeks. One of the items assesses General Health and another item assesses Overall Quality of Life. The rest of the items are grouped into four domains: 1-physical health (7 items), 2-psychological relationships (6 items), 3-social relationships (3 items), and 4-environment (8 items). Mean substitution is used to create raw domain scores with missing data, but raw domain scores are not created if more than 20% of the items have not been completed. The raw domain scores are standardized so that the total score for each domain ranges from 4 to 20 where the highest score indicates a better quality of life for that domain. The WHOQOL-BREF presented adequate psychometric properties, showing a Cronbach’s alpha value of 0.9 [20].

The Satisfaction with Life Scale [21] is an instrument which evaluates individuals’ satisfaction related to various aspects of life. The instrument was originally developed to measure University students’ satisfaction with life. We have modified the scale by removing the question pertaining to satisfaction with student life. The modified scale (SLS-6) has 6 items measuring satisfaction with life in general, physical wellbeing, psychological wellbeing, social relations, leisure activities, and satisfaction with the financial situation. Each item is scored on an eleven-point scale ranging from 0 points (totally unsatisfied with life) to 10 points (totally satisfied with life). The SLS-6 presented satisfactory psychometric properties, with a Cronbach’s alpha of 0.8 and internal validity values ranging from 0.4 to 0.7 [21].

The six-point Likert scale Patient Based Global Impression of Severity Scale (PGIS) [22] is a single item scale previously used in research studies to measure patients’ severity of their physical health condition on a scale ranging from ‘not ill at all’ to ‘extremely ill’. The PGIS has excellent construct validity and has been widely used in studies of chronic diseases [22].

Based on research team experience in previous validation studies [13, 14], a median time of 40 min to answer all the scales per participant was expected. Those participants who have other LTCs than PD were asked to complete the scales regarding PD as the most influencing condition in their lives.

Data collection was conducted in the period between December 2020 and February 2022. Due to the COVID-19 pandemic, face to face data collection was modified to email, post or on the telephone. To homogenise the data collection process and minimise potential random bias, the following standardized protocol was established: interested participants contacted the research team via email or telephone, in response to the advertisement. The interested individuals fulfilling inclusion/exclusion criteria were send (by email or post) a participation information sheet regarding the study, a consent form, and the questionnaires. Those completing via email were required to download the documents, complete, upload and return them, those receiving via post completed the documents and returned them using a ‘Freepost’ address provided by the study team. If the participant wished to complete the documents over the telephone, then the questionnaires were sent via post or email prior to the phone ‘appointment’, during the appointment, the researchers’ role was only to explain the study and ensure all questions were fully completed. If the questionnaires were not returned within 3 weeks a one-time reminder email or telephone call was made. At the end of data collection, each participant was provided with a 10-pound ‘thank you’ voucher.

### Data analysis

Data formatting and analysis was undertaken with the software packages SPSS (IBM Corp. Released 2020. IBM SPSS Statistics for Windows, Version 27.0. Armonk, NY: IBM Corp), SAS9.4 (Cary, NC: SAS Institute Inc.), and R studio (RStudio Team, 2020. RStudio: Integrated Development for R. RStudio, PBC, Boston, MA URL (http://www.rstudio.com/). Descriptive statistics, such as central tendency measures and proportions, were used to analyse sociodemographic characteristics of the sample and also PD related information.

Considering that living with LTCs like PD is a complex phenomenon that could not be simplified to just inner-processes (i.e., coping, integration, adjustment) [9], analyses for the total LwLTCs scale were conducted. The following psychometric properties of the LwLTCs-PD were tested:

- Feasibility and acceptability. Quality of data was considered satisfactory if missing data was < 5% [23]. Floor and ceiling effect were deemed acceptable if they were < 15% [24] and the skewness was expected between − 1 to + 1 [25].
- Internal consistency. The LwLTCs scale consists of 26 Likert scale items, the domain scores are not normally distributed (significance of Shapiro-Wilk test for normal distribution < 0.05), hence internal consistency of the LwLTCs total score and domain scores was quantified in form of inter-item polychoric correlations and ordinal alpha [26]. For research purposes an alpha value higher than 0.70 was recommended [27]. Inter-item correlations in the form of Spearman rank correlations were determined.
- Reproducibility (test-retest). This analysis was evaluated through a second administration of the questionnaire to a subset of 50 participants within a timespan of 7–10 days after the baseline assessment [28]. Reproducibility of domain and total scale score was determined using intraclass correlation coefficient (one way, random effect, ICC) with ICC values ≥ 0.75 being acceptable [29]. A sample of 50 participants was calculated to estimate the ICC to within +/-0.1 if ICC = 0.8 and to within +/-0.05 if ICC = 0.9. The LwLTCs-PD scale and the PGIS were used to determine the stability of participants’ health status. Reproducibility of LwLTCs scale items were determined by calculating Cohen’s kappa with values of > 0.75 considered as moderate level of reproducibility [30].
- Content validity, was established through a *bespoke* questionnaire related to the LwLTC Scale and the PPI group [15].
- Construct validity was assessed with Spearman rank correlations by determining (1) convergent validity: a moderate (r_s_ ≥0.35–0.50) or strong relationship (r_s_ >0.50) was hypothesized between LwLTCs scale and similar constructs measured by DUFSS, SLS-6, and WHOQOL-BREF, (2) discriminant validity: a weak (r_s_< 0.30) association was hypothesized between LwLTCs scale and dissimilar constructs such as age at diagnosis, duration of PD, and PGIS.

Internal validity defined as the intercorrelations between the LwLTCs scale domains (standard, r_s_ = 0.30–0.70) [25, 31] was determined.

For known-groups validity, differences in LwLTCs scale scores in the participants grouped by gender, employment, comorbidity, lifestyle changes and therapy were analysed [31, 32]. Kruskal-Wallis statistic and the Mann-Whitney U test were used for groups comparison.

## Results

A total sample of 241 people living with PD were included in this first validation study of the English version of the LwLTC scale. 53.9% (n = 130) of this sample were male, the age of the people living with PD ranged between 42 and 92 years old, most (n = 237, 98.3%) participants reported white ethnicity, 76.8% (n = 185) were married, and many were fully retired (79.3%, n = 191). Regarding education level of these people living with PD, 29.5% (n = 71) had a University degree, 24.9% (n = 60) had college degree, and 19.9% (n = 48) completed post graduate studies. Further sociodemographic characteristics of the sample are shown in Table 1.

**Table 1:** Sociodemographic characteristics of the sample and PD related information

Demographic variables	Response options	Total individuals living with PD N (%)
Gender	Male	130 (53.9%)
	Female	110 (45.6%)
Ethnicity	White	237 (98.3%)
	Mixed or multiple ethnic groups	1 (0.4%)
	Asian or Asian British	3 (1.2%)
Marital status	Married	185 (76.8%)
	Widowed	20 (8.3%)
	Living with partner	12 (5%)
	Single	7 (2.9%)
	Separated/divorced	15 (6.2%)
	Other	1 (0.4%)
Educational level	Primary school	0%
	Secondary school	39 (16.2%)
	Apprenticeship	9 (3.7%)
	College	60 (24.9%)
	University degree	71 (29.5%)
	Post graduate studies	48 (19.9%)
	Doctorate	12 (5%)
Employment	Retired	191 (79.3%)
	Employed (> 40 h)	11 (4.6%)
	Employed (< 40 h)	19 (7.9%)
	Looking for work	1 (0.4%)
	Not employed and not looking for work	9 (3.7%)
	Disabled/ not able to work	10 (4.1%)
Rural/ Urban	Area with < 2500 habitants	52 (21.6%)
	Area between 2501 and 10,000 habitants	68 (28.2%)
	Area > 10,000 habitants	118 (49%)
	Other	3 (1.2%)
Household income	< £29,400	65 (27%)
	Around £29,400	55 (22.8%)
	> £29,400	118 (49%)
Co-morbidity	None	82 (34%)
	Arthritis	125 (51.9%)
	Parkinson’s disease	27 (11.2%)
Medication	Yes	221 (91.7%)
	No	19 (7.9%)
Surgery	Yes	12 (5%)
	No	229 (95%)
Therapy	Yes	94 (39%)
	No	147 (61%)
	**Range**	**Mean (Standard Deviation)**
Age	42–92	66.8 ± 8.3
Age PD diagnosis	29–84	59.6 ± 9.1
Duration PD	1–40	7.5 ± 5.9

PD: Parkinson’s disease

Although participants were recruited based on having PD, over half of the sample (51.9%, n = 125) also had other LTCs, such as arthritis. The duration of PD was between 1 and 40 years (7.5 ± 5.9) where almost all the participants (91.7%, n = 221) had received specific medication for PD and had not had surgery due to PD (95%, n = 229). See Table 1 for further PD related information.

### Feasibility and acceptability of the LwLTCs scale in English-speaking PD population

Results related to feasibility of the LwLTCs scale, showed that 6 individuals did not complete 1 or 2 items on the scale. Three participants failed to complete an item in domains 2-Coping or 5-Adjustment, and one participant failed to complete one item in domain 3-Self-management. Regarding acceptability of the LwLTCs scale, floor and ceiling effects were both < 15% and skewness values were between – 1 and + 1 for and the total score of the LwLTCs scale. See Table 2 for further information.

**Table 2:** Feasibility/ acceptability, reliability, and precision of the LwLTCs scale within English Parkinson’s disease population

	LwLTCs-PD scale					
	**Domain 1-** **Acceptance**	**Domain 2- Coping**	**Domain 3- Self-management**	**Domain 4- Integration**	**Domain 5- Adjustment**	**Total** **score**
Data Quality (n missing values)	0	3	1	0	3	6
Floor effect (%)	0.4	0	0	0	0.4	0
Ceiling effect (%)	3.7	1.7	3.7	6.2	2.5	0
Skewness	-0.58	-0.26	-0.52	-0.56	0.09	-0.27
Ordinal alpha	0.77	0.73	0.59	0.64	0.85	0.89
Polychoric item-item correlation	0.29–0.67	0.16–0.60	0.28–0.45	-0.05–0.63	0.30–0.77	-0.05–0.77
Reproducibility (ICC)	0.85	0.86	0.62	0.67	0.81	0.88

LwLTCs scale: Living with long term conditions; ICC: Intraclass correlation coefficient

### Internal consistency of the LwLTCs scale in English-speaking PD population

Results related to internal consistency of the LwLTCs scale showed that ordinal alpha was 0.89 for the total scale and for the domains ranged between 0.59 (domain 3-Self-management) and 0.85 (domain 5-Adjustment). All inter-item correlations were higher than established standard value, except for domain 4-Integration. See Table 2 for further information.

### Reproducibility of the LwLTCs scale in English-speaking PD population

This analysis was determined on 50 consecutive participants living with PD. The ICC for the total scale was 0.88 and for all domains was over 0.75 except for domains 3-Self-management and 4-Integration (see Table 2). For items, Cohen’s kappa ranged between 0.13 (item 22) and 0.65 (item 6).

### Content validity and construct validity of the LwLTCs scale in English-speaking PD population

Results related to content validity demonstrated that the LwLTC Scale was useful and could unfold relevant aspects of the person [15]. Wording of some items was improved due to cultural differences. For instance, the word ‘fight’ in item 5 *(I try to cope and fight the disease)* was identified as a negative word or item 25 *(despite the problems the PD creates, I have found new meaning in my life)* was identified as an odd item.

In agreement with our hypothesis the results in Table 3 showed that the LwLTCs scale is strongly correlated with SLS-6 (r_s_=0.67) and WHOQOL-BREF (r_s_=0.54), and moderately correlated with DUFSS (r_s_=0.45). As hypothesized all the dimensions of SLS-6 showed strong correlations with LwLTCs scale, except the domain related to the financial situation which showed weak correlation (r_s_=0.24). In addition, the weak or negligible correlations that were hypothesized between LwLTCs scale and PD related information, such as patient impression of PD severity (PGIS) or duration of PD (see Table 3) were also present.

**Table 3:** Construct and internal validity of English version of the LwLTCs scale

		LwLTCs-PD scale					
		Domain 1- Acceptance	Domain 2- Coping	Domain 3- Self-management	Domain 4- Integration	Domain 5- Adjustment	Total score
**Convergent validity**	**Age**	0.10	-0.06	-0.08	0.06	-0.08	-0.03
	**Age PD diagnosis**	0.14*	-0.05	-0.05	0.09	-0.07	0.001
	**PD duration**	-0.10	0.03	0.02	-0.06	0.01	-0.02
	**PGIS**	-0.30**	-0.21**	-0.20**	-0.35**	-0.28**	-0.36**
	**DUFSS**	0.31**	0.43**	0.27**	0.39**	0.28**	0.45**
	**SLS-6**	0.49**	0.49**	0.26**	0.55**	0.59**	0.67**
	Satisfaction - Physical health	0.46**	0.32**	0.21**	0.49**	0.49***	0.55**
	Satisfaction - Psychological well-being	0.44**	0.45**	0.28**	0.51**	0.44**	0.59**
	Satisfaction - Social relations	0.31**	0.49**	0.25**	0.43**	0.37**	0.51**
	Satisfaction - Leisure	0.38**	0.42**	0.26**	0.54**	0.51**	0.58**
	Satisfaction - Financial situation	0.13*	0.14*	0.21**	0.25**	0.18**	0.24**
	**WHOQOL-BREF**	0.39**	0.41**	0.29**	0.53**	0.42**	0.54**
	WHOQOL-BREF - Physical Health	0.41**	0.32**	0.21**	0.51**	0.35***	0.47**
	WHOQOL-BREF - Psychological Health	0.56**	0.51**	0.37**	0.64**	0.51***	0.69**
	WHOQOL-BREF - Social relationships	0.30**	0.45**	0.27**	0.43**	0.38**	0.50**
	WHOQOL-BREF - Environmental	0.39**	0.44**	0.40**	0.54**	0.34**	0.54**
**Internal validity**	**Domain 2-Coping**	0.33***	-	-	-	-	-
	**Domain 3-Self-management**	0.13*	0.45***	-	-	-	-
	**Domain 4-Integration**	0.40***	0.52***	0.48***	-	-	-
	**Domain 5-Adjustment**	0.33***	0.60***	0.44***	0.47	-	-

LwLTCs scale: Living with long term conditions; PD: Parkinson’s disease; DUFSS: The Duke-UNC Functional Social Support Questionnaire; SLS-6: Modified Satisfaction with Life Scale; WHOQOL-BREF: The World Health Organization Quality of Life Instrument-Brief

Spearman rank correlations ***p < 0.001;**p < 0.01; * p < 0.05

According to internal validity analysis, correlation values between LwLTCs domains ranged from 0.44 to 0.60, except for domain 1-Acceptance that showed < 0.40 correlation coefficients with the other domains. See Table 3 for further detail.

As showed in Table 4, the total score of known-group validity analysis showed statistically significant difference just for therapy and co-morbidity (p < 0.05), yet no significant differences were identified for gender, employment situation, or lifestyle changes.

**Table 4:** Known-groups validity

Variable	Categories	LwLTCs-PD scale	*p* value
Gender	Male	68.50 ± 14.39	0.43*
	Female	66.60 ± 14.63	
Employment	Employed	69.77 ± 12.23	0.50**
	Unemployed	64.10 ± 19.99	
	Retired/disabled	67.63 ± 14.57	
Co-morbidity	None	69.96 ± 14.49	0.03**
	Arthritis	65.79 ± 14.32	
Lifestyle changes	Yes	67.09 ± 13.40	0.44*
	No	68.19 ± 15.63	
Therapy	Yes	64.95 ± 15.18	0.03*
	No	69.32 ± 13.79	

LwLTCs scale: Living with long term conditions scale

* Mann-Whitney test

** Kruskal-Wallis test

## Discussion

This is an innovative and pioneering study in the UK because it is the first time that the LwLTCs scale is tested among an English-speaking population. Mirroring previous LwLTCs scale validation studies carried out in Spanish-speaking population, the aim of this study was to analyse the psychometric properties of the LwLTCs in individuals living with PD in the UK. The scale was applied to a wide and representative PD population (n = 241) that present diverse sociodemographic characteristics, such as wide age range, ethnicity, or employment situation, among others. Moreover, participants were recruited from different community care services in England which supports the consistency of the results at least for this cultural and linguistic setting. Therefore, although the sample size of this validation study is modest, the sociodemographic characteristics of the sample allow to generalize the results to, at least, a PD population living in the UK.

Overall, the analysed psychometric properties for the total scale were deemed satisfactory. Considering feasibility and acceptability of the LwLTCs scale, the results were excellent. Quality of data was satisfactory with only 6 missing values. Hence, 97.51% of the cases were computable probably due to the close follow-up process conducted by the researchers during the data collection procedure and the clear steps established in the protocol. Acceptability of the total LwLTCs scale was satisfactory showing that the scale does not present floor nor ceiling effects and that skewness values were into established values. Hence, we could state that, as a whole, the total LwLTCs scale covers the full spectrum of the construct ‘living with LTCs’.

Reproducibility of the LwLTCs-PD scale was excellent with an ICC value over standard values. However, when analysing the domains, domain 3-Self-management and domain 4-Integration showed ICC values slightly lower than expected. Internal consistency for the total LwLTCs scale was excellent (ordinary alpha = 0.89) reflecting the satisfactory intercorrelations between items on the scale. However, the internal consistency of domain 3-Self-management and domain 4-Integration were under the limit of stipulated value (0.7). This finding for domain 3-Self management is a recurrent result identified in previous LwLTCs scale studies with a Spanish speaking PD population [13, 14]. Nevertheless, none of the previous Spanish validation studies were conducted in individuals with other LTCs [33], such as COPD [34], chronic heart failure [35], diabetes mellitus type 2 [36] or osteoarthritis [37] showed this limitation in domain 3-Self-management. It needs to be investigated if the items grouped in domain 3-Self-management have redundant content specifically for individuals living with PD but not for other LTCs. The lack of internal consistency found in domain 4-Integration, might be due to item 18. This item showed low inter-item correlation with the other items included in domain 4 and internal consistency level of the domain will increase to acceptable levels (0.76) if this item was removed from the scale. Therefore, confirmatory factor analysis (CFA) using Unweighted Least Square estimation [38] was performed to validate the 5-domain structure of the LwLCTs scale (supplementary material). The findings of the CFA suggested that item 18 should be removed, and items 10 and 11, and 23 and 24 overlap, indicating the need to include just one of those items. Hence, based on the CFA a 23-item 5 domain structure could be a better fit for the data than the originally proposed 26-item 5 domain structure for the scale. These findings are consistent with previous findings in people with diabetes mellitus type 2 [36]. However, sample size is not big enough to run a CFA for this study. Therefore, a bigger sample size is recommended for future validation studies in order to run CFA.

Overall, it could be concluded that the LwLTCs scale is a valid scale to evaluate how the person is living with PD. However, future validation studies to prove the repeatability of the total scale and particularly, domains 3-Self-management and 4-Integration in other LTCs is recommended. In addition, the internal consistency findings identified in this study will also be carefully considered when developing further studies on the English version of the LwLTC in populations with other LTCs.

Construct validity results of the LwLTCs-PD scale were as expected and almost a replicate of previous Spanish-speaking population validation studies results. The construct “Living with LTCs” and particularly, living with PD presents several parallelisms between countries. As identified in our previous Spanish-speaking studies, living with PD is highly correlated with constructs related to the person’s experience and not the disease, per se [7]. In this sense, the expected hypotheses for convergent validity were satisfactorily achieved where the LwLTCs scale showed strong correlations with WHOQOL-BREF and SLS-6 and moderate correlations with DUFSS. Regarding correlations between LwLTCs scale and DUFSS, this result was not surprising as a recently conducted empirical study [7] showed that social support and satisfaction with life are factors that significantly influence in living with PD. Concretely, this finding is not new in the literature, where social support is presented as a star factor when living with PD [7, 39, 40]. Similarly, a strong correlation between the LwLTCs scale and SLS-6, was also expected. Previous studies conducted in PD population [7, 41] confirmed this direct and significant correlation between satisfaction with life and living with PD. Satisfaction with financial situation is the only dimension from the SLS-6 that did not correlate with the LwLTCs scale. This is an interesting result which would need further exploration among PD population and other LTCs in the UK. Following the established hypothesis, correlations between the LwLTCs scale and WHOQOL-BREF were also as expected. Other works in this field [3, 8, 9] also highlighted the strong correlation between living with PD and quality of life. Concretely, based on an in-depth conceptual analysis [9] quality of life was identified as clear consequence of the daily living. Therefore, considering these findings, once again we could highlight the need to place the emphasis on the person and in his/her daily living with an LTCs such as PD, and not just on the disease as it is frequently done in current clinical practice. It is necessary to incorporate multidisciplinary and individualised interventions in nowadays health and social services, focusing on the factors that directly influence in living with PD, as for social support, satisfaction with life, or quality of life. We really advocate the necessity to develop person-centred interventions or individualized care pathways, incorporating nonpharmacological or PD-specific measures that address the factors that are paramount in the daily living with PD. Consequently, possible negative aspects of the daily living with an LTC, such as lack of support, loneliness, poor quality of life or dissatisfaction with life, could be prevented, and a more positive living achieved. Therefore, PD programmes that mobilise and optimise the use of community resources, and increase personal networks and social support seem to be the direction to approach neurodegenerative conditions as PD.

Regarding internal validity of LwLTCs scale, satisfactory results were identified for all domains, except for domain 1-Acceptance (0.13). According to previous in-depth conceptual as well as empirical analyses conducted in the last decade to achieve a better understanding of the concept ‘Living with an LTC’ [7–10] and based on results emerged in this study, once again we could conclude that domain 1-Acceptance is always the key and starting point to achieve a positive living with PD. Interestingly, while other domains could not be necessary, acceptance is always the first and essential process to achieve a positive living with PD. Acceptance is comprised as an internal, illness-independent process that allows the person to understand and assume the reality [9]. Therefore, only when the person has accepted the diagnosis of PD, and thus the new situation, can he/she move forward towards a positive living.

Finally, known-groups validity showed that the LwLTCs scale, does not discriminate depending on gender, employment situation, or lifestyle changes. This indicates that the scale evaluates in an equal manner the degree of living with PD independently of gender, employment situation, or lifestyle changes. Presently, we could conclude that the LwLTCs scale is a useful instrument to be used with a diverse sample of people living with PD without differentiating their gender, employment situation, or lifestyle changes. Nowadays, it is very important to have available and comprehensive clinical instruments like the LwLTCs scale, that could be used in a heterogenous sample capturing individuals’ needs with different sociodemographic characteristics. For instance, the LwLTCs scale could become very useful for people with lower socio-economic and cultural resources.

This English version of the LwLTCs scale could be used as a complement to conventional generic health-related quality of life measures and as a basis for evaluation where interventions may affect both health and social care outcomes. In this sense, using the LwLTCs scale in routine care for people living with PD, could result in more optimal health care utilization without sacrificing quality of life and economic costs; adoption could ensure effective risk stratification and early identification of people with greater complex care coordination need and at high risk of poor ability to self-manage. Specifically, the implementation of the LwLTCs scale is intended to inform person-centred care pathways and more effective multidisciplinary and multisectoral referral processes to personalise and meet individual needs [9].

The findings presented here should be viewed considering some limitations. Firstly, the inclusion of just one English-speaking country (England) in this study could be seen as a limitation related to external validation. Although we included a wide and heterogeneous sample living with PD, the inclusion of further English-speaking populations from other countries like Australia or the US is recommended for further validation studies. However, the LwLTCs scale was translated to English language and therefore, it is ready for future collaboration studies in other English-speaking countries. In addition, the sample size could be slightly insufficient to conduct some statistical calculations, such as confirmatory factor analysis. Therefore, for future LwLTCs scale validation studies, a larger sample size is recommended. Besides, individuals living with PD and cognitive deterioration were excluded from the study. The main reason was to ensure that individuals answer the scales in a self-reported manner and in a conscious way without any cognitive difficulty. Moreover, some of the data collection was conducted via telephone appointment which could have influenced the participant’s response. However, following established recruitment protocol, the researcher just explained the study and read the items of the scales, without additional explanation. Besides, recruiting through websites and social media might be a limitation as all PD population might not be able to access internet-based technologies. Finally, we should also mention that this validation study has been carried out during the COVID-19 pandemic, which has increased the difficulty of accessing people living with PD. Besides, COVID-19 lockdown has been a challenge to test the applicability of the scale remotely, via email or telephone interview.

This study also presents several strengths such as heterogeneity of the sample, inclusion of individuals living with multiple LTCs and not just PD, data collection carried out in health but also social care settings, and finally, although mentioned as a weakness, COVID-19 pandemic has become a positive opportunity to test the applicability and versatility of the scale as well as teamwork in circumstances that were not expected.

## Conclusions

This first validation study of the LwLTCs scale in English-speaking PD population showed satisfactory psychometric properties. Therefore, with caution the LwLTCs scale is ready to be used in clinical practice and research in English-speaking population living with PD. In this regard, future implementation and validation studies are recommended to analyse how the scale could apply to other LTCs population, which are now in progress.

## Electronic supplementary material

Below is the link to the electronic supplementary material.

Supplementary Material 1

## Data Availability

The datasets used and/or analysed during the current study are available from the corresponding author on reasonable request.

## Abbreviations

LTCs: Long term conditions
PD: Parkinson’s disease
LwLTCs: Living with Long term conditions
