# Supplementary figures and images for: First validation study of the living with long term conditions scale (LwLTCs) among English-speaking population living with Parkinson’s disease
Source: Health Qual Life Outcomes. 2023 Jul 10;21:69. doi: 10.1186/s12955-023-02154-6 (PMC10334560; doi:10.1186/s12955-023-02154-6)

**
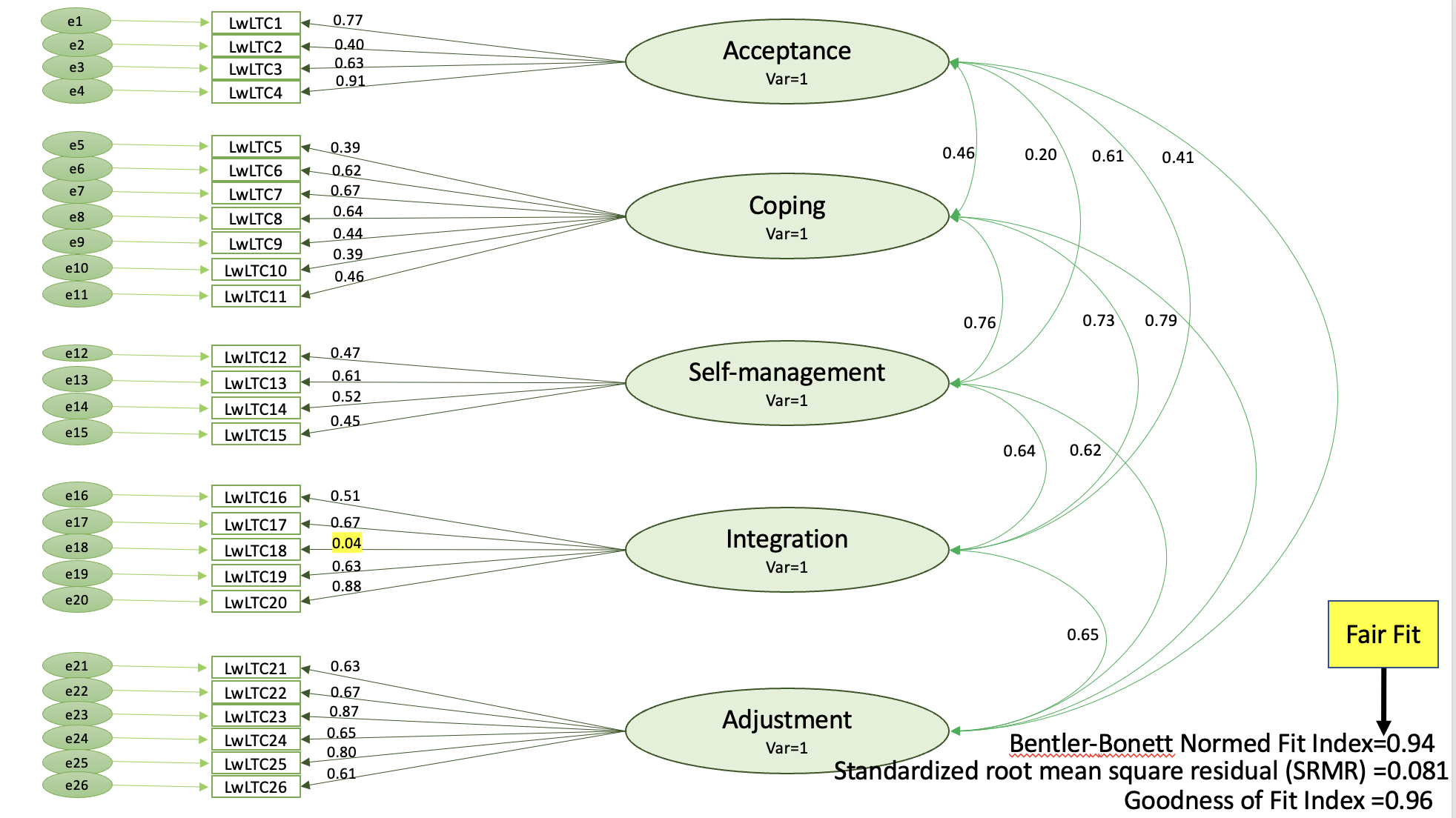
Supplementary material. Confirmatory factor analysis**

Supplement: Supplementary file 1 — Supplementary Material 1 [file 12955_2023_2154_MOESM1_ESM.docx]
